# Time perspectives and procrastination in university students: exploring the moderating role of basic psychological need satisfaction

**DOI:** 10.1186/s40359-023-01494-8

**Published:** 2024-01-02

**Authors:** Nuria Codina, Isabel Castillo, José Vicente Pestana, Rafael Valenzuela

**Affiliations:** 1 Department of Social Psychology and Quantitative Psychology, University of Barcelona, Pg. de La Vall d’Hebron 171, 08035 Barcelona, Catalonia Spain; 2 Department of Social Psychology, University of Valencia, Av. Blasco Ibáñez 21, 46010 Valencia, Valencia Spain; 3 Serra Húnter Programme, Barcelona, Spain

**Keywords:** Time perspectives, Future time perspective, Procrastination, Implemental delay, Basic psychological need satisfaction, Lateness

## Abstract

**Background:**

Research on procrastination, regarding time perspective factors and basic psychological need satisfaction (BPNS) has placed this problem at the meeting point of individual and contextual variables. The present study focused both on the individual, given that time perspectives can be defined as a person’s attitude to an object (time) at three moments (present, past, future); and on contextual aspects, because the satisfaction of basic psychological needs (competence, autonomy, relatedness) is facilitated or made difficult by social contexts. Based on this, the aim of this study was to analyse the relationships between time perspectives and inter-subject procrastination variations, testing the moderating role of BPNS in this relationship.

**Method:**

A total of 1,188 undergraduate students, aged 17–50 years (*M* = 20.02, *SD* = 2.63), completed three questionnaires containing the variables of interest.

**Results:**

Regression analyses showed significant negative (thus, potentially protective) association of future time perspective with all three procrastination dimensions (decisional procrastination, implemental delay, and lateness). Conversely, past-negative time perspective showed a positive (thus, potentially adverse) association with procrastination. Satisfaction of the need for competence also showed a negative (thus, potentially protective) association with all procrastination dimensions. On eight occasions, the relationships between time perspectives and procrastination dimensions were moderated by psychological need satisfaction.

**Conclusions:**

These findings show that BPNS may play relevant roles in the negative (favourable) relationships between procrastination dimensions and positive time perspectives, as well as in the positive (adverse) associations between negative time perspectives and procrastination dimensions. Contextual interventions fostering enhanced levels of perceived autonomy, competence, and relatedness, as well as future time perspective, are thus strong candidates to consider for use and evaluation by policy makers, pedagogues, teachers, coaches and other professionals interested in counteracting procrastination tendencies.

## Introduction

Procrastination in university students, with an incidence of between 80 and 95% [1], is a problem observed in different areas of everyday life [2] and it subsequently affects graduates’ performance levels and well-being in the work domain [3]. These figures alone call for research into how to alleviate or reduce procrastinating behaviours and their negative consequences. Research oriented towards this objective has tried to determine the nature of this problem, as well as to analyse its relationships with other constructs related to attitudes towards time (in particular, their balance or imbalance) and the contexts that favour self-determination (in terms of autonomy, competence and relatedness).

### Procrastination, trait and state

Research and intervention around procrastination require a thorough consideration of the possibilities and limits conveyed by the nature and characteristics of the construct. In this sense, depending on how procrastination is conceived of, alternative methods of investigation and intervention can be explored. In 1985, the American Psychological Association (APA) stated that procrastination was to be understood as a “habitual, often counterproductive postponement” [4], in this way underscoring that frequency of postponement can originate habitual postponement. Later on, its volitional character was posited, signalling that “to procrastinate is to voluntarily delay an intended course of action despite expecting to be worse off for the delay” (p. 66) [5]. Within this conception, the individual is thought of as the protagonist of procrastination dynamics. Furthermore, two other conceptions have emerged that consider the procrastination: as a trait [6] and as a state [7]. These two conceptions have both found empirical support, both showing explanatory potential, thus, at the same time suggesting that the phenomenon of procrastination could admit complementary – even if diverse – explanations.

Regarding procrastination as a trait, it has been evidenced – among other ways – through the adverse influences of stable procrastination tendencies on health and well-being [8], including troublesome impacts on the health of possible future selves [6]. Regarding procrastination as a state, – among other aspects – it has been observed in the fluctuations in procrastination over time and across different situations [9, 10]. In sum, evidence for both approaches suggest that “procrastination appears as an individual tendency that can be influenced by certain contexts” [2]. Thus, not everyone is a procrastinator but everyone procrastinates at some time [11] or, in other words, both trait procrastination (linked with individual differences) and state procrastination (linked with contextual elements such as tasks) need to be considered. With regard to this dual conception of procrastination – and by way of contributing knowledge enriching the discussion on how to alleviate and reduce it – this study assesses two issues in particular: the associations of time frames with procrastination [11, 12] and the possible moderating role of basic psychological need satisfaction (BPNS) on the aforementioned relationships.

### Time frames and procrastination

The time frames related to procrastination bring to light individual differences with respect to the perception of the passage of time, making it possible to discern the degree to which people are competent in time management [13]. In this regard, the time perspectives proposed and developed by Zimbardo & Boyd [14] deserve special attention. They distinguish five perspectives in the general attitudes towards time (present-hedonistic, present-fatalistic, past-positive, past-negative, and future).

The results obtained from different studies show that the predominance of one perspective or another can be linked to both more or less healthy habits and risk behaviours [15]. In this sense, it has been questioned whether there is a “good” or “positive” combination of time perspectives – and, contrariwise, a “bad” or “negative” combination, and these are respectively referred to as the balanced time perspective and the deviation from balanced time perspective [16]. Generally speaking, a balanced time perspective (BTP) is characterized by a combination of lower levels of past-negative and present-fatalistic factors, higher scores in the positive past and future, and moderate levels of hedonistic present. The opposite is true for the deviation from balanced time perspective (DBTP), which results in poorer well-being and difficulties in adaptation. Depending on how these factors are combined with each other and whether or not a balance between them can be observed makes these relationships particularly important. And this is fundamental to well-being and, by extension, health [17].

Regarding the relationships between these perspectives and procrastination [3, 12], the main results show that procrastination is associated with time perspectives. Particularly, future time perspective has been found to be negatively and moderately associated with procrastination, whereas both present time perspectives have been found to be positively though weakly associated with this problem [18]. In accordance with these results, it is necessary to further explore these relationships between time perspectives and procrastination, taking into account the links between this problem, the balanced time perspective and its deviation – about which, to our knowledge, little is understood.

### Contextual influences and procrastination

From a state procrastination perspective, it has been argued that procrastination tends to become habitual when a person frequently avoids certain aversive tasks considered difficult or tedious [19] (Pychyl et al., 2000), leading to self-regulatory failures that come from not being able to do things as planned [5]. Contrarily, when, for instance, adolescents feel self-determined (this is, autonomous and competent), they may regulate their learning efforts better and procrastinate less [20]. Basic psychological needs theory [21], a mini-theory of Self-determination Theory (SDT) postulates that contexts within which a person feels competent, autonomous, and related tend to aid self-regulation [22], limiting the potential for self-regulation failures such as procrastination. These three conditions for high quality motivation are denominated as basic psychological need satisfaction (BPNS) and distinguishes three universal needs [22]:

- *Autonomy* (rather than independence) is the ability to endorse one’s own actions (even if following others). Thus, the need for autonomy is satisfied when people are able to choose activities they personally value or intrinsically enjoy, and when they have relative control over the ways in which they carry them out.
- *Competence* is the perception of being able to complete a given task under specific contextual circumstances. Thus, the need for competence is satisfied when people feel they have the ability to complete the given task.
- *Relatedness* is the need satisfaction that can be described as not feeling isolated from (i.e., connected to) the people with whom an individual shares specific activities.

According to Ryan & Deci [22], whether these basic psychological needs are satisfied or thwarted depends on contextual factors and task-related conditions and, in turn, this explains the resulting quality of motivation and may have a negative or positive influence on wellbeing. Specifically, it has been argued that people across cultures, independently of whether they want it or not, require sufficient satisfaction of the aforementioned basic psychological needs in order to thrive in what they do.

In relation to procrastination, it has been shown that Basic psychological need satisfaction (BPNS) – such as satisfaction of the need for competence – plays a mediating role in the relationship between social context and procrastination [23]. In fact, when the perceived style of the teacher in the classroom is autonomy-supportive, higher levels of satisfaction of the need for competence are observed and also less procrastination. In line with this, perceived competence has been found to have a significant negative relationship with procrastination, and perceived autonomy has been found to be indirectly and negatively associated with procrastination, via the mediating role of intrinsic motivation [24]. As for the previous, rather scant evidence has been offered by studies of BPNS in relation to procrastination and time perspectives, for instance, it has been found that deviation from balanced time perspective (DBPT) may play a moderating role in the relationship between BPNS and trait anxiety [25], with BPNS in turn mediating between social support and procrastination [26].

Furthermore, BPNS moderates the relationships between different behaviours and psychological aspects. For example, BPNS moderates the relationships between future aspirations and outcomes related to well-being, such as meaning in life. In fact, life goals that facilitate increased BPNS have been found to better promote meaning and well-being [22]. By way of examples, BPNS can buffer the effects of negative life events [27], moderate the adverse effects of job demands on turnover intentions [28], and enhance the favourable effects of physical activity on positive feelings [29].

As regards, the relationships between time perspectives, BPNS has been found to be negatively associated with negative time perspectives (past negative, present fatalistic) and positively associated with positive time perspectives (present hedonistic, past positive, and future); furthermore, from a person-centred perspective, more balanced and less negative time perspective profiles showed higher BPNS [30]. Especially, future time perspective has consistently shown positive relationships with BPNS: In youth athletes, both perceived competence and perceived autonomy were found to be positively associated with future time perspective [20]; and in university students, self-concordant future goals were characterised by higher perceived satisfaction of the basic psychological needs for autonomy, competence, and relatedness, as compared to non-self-concordant future goals [31]. Also, from a person-centred perspective, BPNS has been associated positively with a Balanced Time Perspective (BTP) and negatively with a Deviation from Balanced Time Perspective (DBTP) [32]Time Perspectives and Procrastination in University Students: Exploring the Moderating Role of Basic Psychological Need Satisfaction. Importantly, it has been observed that high school students’ academic achievement was positively predicted by the interaction of BPNS and future orientation [33].

These antecedents support the suitability of conjointly studying the time perspectives and BPNS as potential correlates of procrastination variations, which could interact in the deployment of strategies aimed at coping with procrastination. Based on these antecedents – signalling the importance of BPNS – it is relevant to assess whether BPNS could respectively be associated with greater negative (favourable) associations between procrastination and future and past-positive time perspectives and with smaller positive (adverse) associations between procrastination and past negative and (both) present time perspectives. This could provide stronger empirical evidence for the role of self-regulatory processes in time experience [34].

Based on the above mentioned, the following hypotheses were tested in this paper:

- H_1_: positive time perspectives (past-positive, future) and balanced time perspective are negatively associated with procrastination dimensions.
- H_2_: negative time perspectives (present-fatalistic, past-negative) and deviation from balanced time perspective are positively associated with procrastination dimensions.
- H_3_: BPNS is negatively associated with procrastination dimensions.
- H_4_ The relationships between time perspectives and procrastination dimensions are moderated by BPNS. Specifically,
- H_4A:_ The negative relationships between positive time perspectives (future, past positive) and procrastination variations are strengthened by BPNS.
- H_4B:_ The positive relationships between negative and neutral time perspectives (present fatalist, past negative, and present hedonistic) and procrastination variations are weakened by BPNS.

The present contribution integrates previous findings, concepts and basic theories regarding study variables. Constructs of procrastination and time perspectives have been developed building upon cognitive perspectives; whereas, basic psychological need satisfaction has its origins in humanistic views. Both approaches have posited that self-regulation is a critical aspect of adaptive behaviour. On the one hand, cognitive approaches highlight that self-regulatory mechanisms operate through psychological subfunctions that have to be developed to allow that forethought gets translated into incentives and guides for action, given that intentions and desires alone do not suffice (to connect the future with the present) if people cannot influence their own motivation and behaviour. The importance of time frames can be understood following from this definition, which –at the same time– allows the consideration of procrastination as a self-regulatory problem in translating motives into intentions or actions [35], or in transitioning from motivational into volitional phases of behavioural regulation [36]. It is precisely in this profound consideration of various motivational and strategic processes, that it is sensible to integrate the humanistic perspective of SDT, given that lower level of basic psychological need satisfaction can lead to low quality of motivation and self-regulatory problems. In other words, consistently procrastinating could be connected to low satisfaction of basic psychological needs and to subpar management of time frames. Thus, in the context of the present research, self-regulation is conceived of as a complex process that encompasses behavioural, cognitive and also motivational dimensions rooted in human needs of a universal nature.

Advances in the knowledge of procrastination will be obtained if, with the moderating role of BPNS, the positive (adverse) relationships between negative time perspectives and procrastination are weakened, and the negative (favourable) relationships between positive time perspectives and procrastination are strengthened. In sum, integrating time perspectives and BPNS could facilitate the introduction in the classroom of intervention strategies aimed at facilitating conditions contrary to procrastination among current university students – who find themselves at a crucial stage prior to their incorporation into the labour market as professionals.

## Method

### Participants

Using a non-probabilistic convenience sampling method, with the aim of obtaining a spectrum of participants from public and private universities and diverse degrees, *N* = 1,200 undergraduate students were recruited from two Universities and seven degrees in Catalonia, Spain. These universities and degrees were chosen because the authors fulfilled teaching duties at those universities and degrees and were granted access to these participants: one private university (EUNCET, Business School: Business Administration, *n* = 260), and one public university (University of Barcelona: Architecture,* n* = 124; Engineering, *n* = 112; Life Sciences, *n* = 84; Psychology, *n* = 190; Public Relations,* n* = 268; and Sociology, *n* = 162). According to the general statistics of the regional government of Catalonia (available online at https://www.idescat.cat/), the proportions in our sample of students from the private university (14.4%) and the public university (85.4%) were different from the general figures from both universities (respectively, 43.6% and 66.4%). This official registry of the government of Catalonia does not offer detailed information on the students enrolled in the selected degrees, information that was not possible to obtain from the universities studied themselves.

Questionnaires were completed face-to-face through pen-and-paper format during regular class time. Twelve questionnaires were discarded due to defects in the registered information (i.e., zero variance in individuals’ responses, or unfinished questionnaires). Participants were *N* = 1,188 undergraduate students, 427 men and 752 women (nine did not report gender), ranging in age from 17 to 50 years (*M* = 20.02, *SD* = 2.63). The percentages of men (36.7%) and women (63.3%) in our sample were also different from those observed in the entire university population of the universities studied (respectively, 58.9% and 41.1%). The differences of proportions between our participants and the general population of the universities studied made our sample a non-representative one and generalization of conclusions is discouraged based on these findings solely.

A 99% response rate (1,188 / 1,200) was due to the fact that students answered questionnaires in regular class sessions with their regular teachers.

### Instruments: description and analysis

Data were collected with three instruments accompanied by the required demographics. All the items were rated on the same Likert-type scale ranging from 1 (“Does not describe you at all”) to 5 (“Very characteristic of you”). Before describing study variables, the measurement instruments were assessed for validity via confirmatory factor analyses (CFA), using SPSS AMOS version 26. Shortcomings in factor structure robustness were assessed and addressed as explained below, originating final factor structures which were then used to compute descriptive statistics, correlations, regressions and moderations.

#### Time perspectives

The Spanish version [37] of the original 5-factor 56-item Zimbardo Time Perspective Inventory (ZTPI) [14], did not fit the data well. Model fit was better but not yet acceptable (CFI = 0.760; RMSEA = 0.064; PCLOSE = 0.000), when assessed based on the 29-item Spanish short version (ZTPI-29) [38], using Spanish phrasings by [37]. Supplementary exploratory factor analysis (EFA) showed that some items had lower than desirable factor loadings, or even loaded onto multiple factors including different ones from those expected by the theory. Such findings are unsurprising as they have been common when using the ZTPI and some authors have opted for finding short versions of the inventory to gain in parsimony [39]. Consequently, items with sub-par performance were considered for deletion, in order to find a ZTPI short-version with a better fit and a more parsimonious factor structure (Table 1). Given a theory-based factor structure, measurement can be considered improved if equal proportions of variations in a given factor can be explained by a smaller number of items [40], if at least three items are retained per factor [41]. Thus, a central aim was that of retaining at least three items per factor, following the recommendation of a minimum of three or four items per factor [42]. Even though it is common to retain items with factor loadings greater than 0.30 or 0.40 [43–45], and a cut-off of 0.32 has been argued to be sufficient [46], it was decided to retain items if they achieved a benchmark factor loading of 0.5 or above in their expected factor, because this is argued to be required for practical significance [47]. In the case of one factor (future time perspective), a less stringent cut-off of at least 0.4 or above was used in order to retain a 3-item factor [48–50]. Items not retained in their theoretical factors were the following (original numberings in the 56-item / 29-item versions): Future, 30/17 (*Antes de tomar una decisión, evalúo los costos y beneficios* / “Before reaching a decision I assess costs and benefits”) [reflects decision making and cost–benefit assessment, rather than future time perspective], 13/06 (*Preparar el trabajo para el día siguiente y cumplir con los plazos es más importante que la diversión de hoy a la noche* / “Preparing work for the next day and meeting deadlines is more important than fun tonight”) [reflects discipline or emotion management, rather than FTP], 18/10 (*Me molesta mucho llegar tarde a mis citas y compromisos* / “It bothers me a lot to be late for my appointments and commitments”) [reflects perfectionism rather than FTP]; past-positive, 02/not available (*Las imágenes, sonidos y olores de la infancia traen recuerdos maravillosos* / “The sights, sounds, and smells of childhood bring back wonderful memories”) [reflects recall of positive events rather than positive evaluation of past], 15/08 (*Me divierten las historias sobre cómo eran las cosas en los “viejos tiempos* / “I'm amused by stories about how things were in the ‘old days’”) [does not determine a positive valence of past perspective, as in sarcasm], 25/14 (*El pasado tiene tantos recuerdos desagradables que prefiero no pensar en ellos* / “The past has so many unpleasant memories that I prefer not to think about them”) [reflects recall of negative events rather than negative evaluation of past], 49/28 (*Me gustan las tradiciones familiares que se repiten regularmente* / “I like family traditions that are repeated regularly”) [reflects preference for family tradition rather than positive past perspective]; present-hedonistic, 08/03 (*Hago cosas impulsivamente* / “I do things impulsively”) [reflects impulsiveness rather than enjoyable experience], 19/11 (*Idealmente, viviría cada día como si fuese el ultimo* / “Ideally, I would live every day as if it would be the last”) [reflects impulsiveness rather than enjoyable experience and is not conceptually distinct from present fatalistic]; present-fatalistic, 37/22 (*Uno no puede planificar el futuro porque las cosas cambian mucho* / “You can't plan for the future because things change so much”) [reflects belief about futility of plans due to change not due to fatalism or predestination]; past-negative, 04/01 (*A menudo pienso que debería haber hecho diferente en mi vida* / “I often think about what I should have done differently in my life”) [does not necessarily reflect -and does not explicitly phrase- a negative evaluation of the past], 36/20 (*Aun cuando estoy disfrutando el presente, tiendo a hacer comparaciones con experiencias similares del pasado* / “Even when I’m enjoying the present, I tend to make comparisons with similar experiences of the past”) [does not necessarily reflect negative evaluation of the past], 54/29 (*Pienso en las cosas buenas que me he perdido en mi vida* / “I think about the good things that I have missed in life”) [does not necessarily reflect negative evaluation of the past].

**Table 1:** Exploratory factor analysis for time perspectives^a^

	Past-negative	Present-hedonistic	Future	Present-fatalistic	Past-positive
ztpi_10			.493		
ztpi_21			.596		
ztpi_40			.621		
ztpi_02					-.527
ztpi_11					-.532
ztpi_20					-.652
ztpi_26		.584			
ztpi_31		.780			
ztpi_42		.840			
ztpi_46		.486			
ztpi_14				.451	
ztpi_38				.527	
ztpi_39				.782	
ztpi_16	.703				
ztpi_27	.468				
ztpi_34	.642				
ztpi_50	.842				

^a^Extraction Method: Maximum Likelihood. Rotation Method: Oblimin with Kaiser Normalization. Rotation converged in 8 iterations

In the past-positive time perspective factor, only two items (11/05, 20/12) met retention criteria. Thus, in order to obtain a three-item factor, we included an item from the original 56-item ZPTI version (not in the 29-item short version), namely item 02/n.a. *(Las imágenes, sonidos y olores de la infancia traen recuerdos maravillosos* / “Familiar childhood sights, sounds, smells often bring back a flood of wonderful memories”), based on its clarity, robust factor loading and the theory-aligned phrasing of a positive evaluation of the past. Lastly, past-negative items 50/25 (*Pienso en las cosas malas que me han ocurrido en el pasado* / “I think about the bad things that have happened to me in the past”) and 54/29 (*Pienso en las cosas buenas que me he perdido en mi vida* / “I think about the good things I've missed in my life”) showed standardized residual covariances greater than 5.0 with other items in the factor. As a result, we alternatively tested deleting one or the other, achieving best fit when deleting item 54/29; this deletion is reasonable given that the deleted item was inversely phrased referring to good things missed, and not bad things that happened, consequently not strictly reflecting a negative evaluation of past events.

Subsequently, in the present study, analysis of time perspectives was based on the resulting five-factor 17-item structure which was considered a more parsimonious instrument and possibly an improved reflective measurement of time perspectives (CFI = 0.913; RMSEA = 0.053; PCLOSE = 0.136). The subscales showed acceptable reliability as determined by the alpha benchmark for factors with few indicators (*α* > 0.60) [51] and its sensitivity to the number of indicators: Future (α_FUT_ = 0.60), past-positive (α_PPO_ = 0.61), and present-fatalistic (α_PRF_ = 0.60) perspectives comprised three items each; whereas present-hedonistic (α_PRH_ = 0.77) and past-negative (α_PNE_ = 0.76) perspectives comprised four items each.

#### Balanced time perspective and deviation from balanced time perspective

Based on the 17-item ZTPI, two integrated measures of time perspective were included in this study, namely balanced time perspective (BTP) and deviation from balanced time perspective (DBTP) [15]. BTP is defined as a favourable combined score, postulated by the authors as high levels of positive perspectives (past-positive and future), low levels of negative perspectives (past-negative and present-fatalistic) and moderately high present-hedonistic perspective [15]. Balanced time perspective was coded as a nominal variable (0,1): answers were coded “1” if they scored 3 or more in both positive time perspectives (past-positive and future), 3 or less in both negative time perspectives (present-fatalistic and past-negative), and not more than 4 in the hedonistic present. In the same vein, following instructions provided by the authors [15], deviation from balanced time perspective was coded as the square root of the sum of squares of the differences between each individual’s scores on each time perspective relative to the optimal score in that same perspective postulated by the authors [11]: future = 4.00, present-hedonistic = 3.90, past-positive = 4.60, past-negative = 1.95, present-fatalistic = 1.50.

#### Basic psychological need satisfaction

BPNS was assessed with the 15-item Spanish version [52] (León et al., 2011) of the *Échelle de Satisfaction des Besoins Psychologiques* [53]. The Spanish language adaptation [54] yielded Cronbach’s alphas of 0.75 for need for autonomy, 0.82 for need for relatedness, and 0.86 for need for competence. CFA revealed that the expected three-factor theoretical model did not fit the data as well as expected (CFI = 0.845; RMSEA = 0.096; PCLOSE = 0.000). The first two items in the autonomy need satisfaction factor failed to load onto their expected dimension (instead, loading onto the competence factor, albeit with low coefficients). A close inspection of their semantic content revealed that these two items had in common that they referred to a perception of trans-situational autonomy or general autonomy, as implied in broad experiences such as “I feel free in my decisions” (01), or “I generally feel free to express my opinions” (04). These two items do not mention the course outline or characteristics, whereas the other three items refer to a perception of autonomy in a more specific domain, as in being able to make students’ opinions heard on course outlines and content, a possibility not available to most participants, that is, situational autonomy or autonomy in academic learning, such as “I have the possibility to make decisions about the subject programs” (07), “I participate in the elaboration of my subject program” (10), or “I can give my opinion on the elaboration of the subjects syllabus” (13). In order to avoid having to delete the first two items, we retained them as a separate factor (Table 2). The model fit the data better when the autonomy factor was split up into two factors (bearing in mind the theory-based distinctions drawn above), one trans-situational and the other situational (respectively, general autonomy and autonomy in academic learning), thereby obtaining a four-factor structure with good fit (CFI = 0.945; RMSEA = 0.058; PCLOSE = 0.007). The reliability of the four subscales was acceptable (α_COM_ = 0.81; α_genaut_ = 0.64; α_acadaut_ = 0.74; α_REL_ = 0.82), as determined by the alpha benchmark for factors with few indicators (*α* > 0.60) [51] and its sensitivity to the number of indicators.

**Table 2:** Exploratory factor analysis for psychological need satisfaction^a^

	Relatedness	Academic autonomy	Comp-etence	General autonomy
pns_01				.585
pns_04				.437
pns_07		.692		
pns_10		.793		
pns_13		.621		
pns_02	.659			
pns_05	.794			
pns_08	.614			
pns_11	.602			
pns_14	.610			
pns_03			-.641	
pns_06			-.706	
pns_09			-.490	
pns_12			-.540	
pns_15			-.847	

^a^Extraction Method: Maximum Likelihood. Rotation Method: Oblimin with Kaiser Normalization. Rotation converged in 12 iterations

#### Procrastination

Procrastination was measured with the Pure Procrastination Scale [55] (PPS: Steel, 2010) whose items come from the General Procrastination Scale [35] (GP: items gp01, gp07, gp09, gp12, and gp19), the Decisional Procrastination Questionnaire [56] (DP: items dp01, dp02, and dp04), and the Adult Inventory of Procrastination [57] (AIP: items aip05, aip09, aip10, and aip15). Spanish language phrasing adaptations used were those of Díaz-Morales et al. (2006) [58], previously tested in [54]. The PPS was expected to yield a three-factor structure (decisional procrastination, implemental delay, and lateness), which it did (CFI = 0.965; RMSEA = 0.044; PCLOSE = 0.907). However, one implemental delay item, stemming from the original General Procrastination Scale [35] (gp01: “I often find myself performing tasks that I had intended to do days before”) yielded a factor loading below 0.30 and was consequently dropped, because it does not reflect ill-being derived from postponement, a definitory feature of procrastination, and also aligned with considerations about a minimum acceptable factor loading of 0.30 [47]. Resulting model fit was equivalent (CFI = 0.966; RMSEA = 0.047; PCLOSE = 0.745), however, a robustness check indicated that the inclusion of said item in the computation of the average of implemental delay led to a decrease in explanation of inter-subject variations in this outcome variable. Furthermore, one decisional procrastination item (dp04: “I delay making decisions until it’s too late”) showed an expectable and theoretically admissible cross-loading with the lateness factor (due to the phrasing including the aspect of being “too late”), which had already been reported previously [59]. The retained 11-item three-factor model of Pure Procrastination (Table 3) showed acceptable reliability (α_DP_ = 0.70; α_ID_ = 0.76; α_LA_ = 0.67; α_PPS_ = 0.83), as determined by the alpha benchmark for factors with few indicators (*α* > 0.60) [51].

**Table 3:** Exploratory factor analysis for pure procrastination^a^

	Implemental delay	Decisional procrastin-ation	Lateness
dp_01		.657	
dp_02		.658	
dp_04		.448	.417
gp_07	.530		
gp_09	.776		
gp_12	.502		
gp_19	.568		
aip_05			.560
aip_09			.581
aip_10			.477
aip_15			.481

^a^Extraction Method: Maximum Likelihood. Rotation Method: Oblimin with Kaiser Normalization. Rotation converged in 8 iterations

### Procedure

Data collection was carried out face-to-face through pen-and-paper format during class time. It was coordinated by researchers from the team, all they with experience in the application of the instruments used. Authorization was obtained from the competent bodies/authorities (i.e., the Heads of the university degree programmes to which the participants belonged), and the teachers whose classes were used for collecting the information. Once in the class with the potential participants, the teacher introduced the researchers, who then outlined the study and asked the students for their voluntary cooperation, including the anonymous use of their answers. The students agreed to participate on an informed, voluntary basis. Questionnaire administration took approximately 25 min.

### Statistical analysis

Statistical analysis performed is consistent with the non-experimental cross-sectional descriptive correlational design used in this research. Descriptive statistics are presented for all study variables (Table 4), also including significant differences in study variables according to BTP (including Cohen’s *d*). Furthermore, we report bivariate correlations among study variables, including DBTP (Table 5), as well as regression analyses of time perspectives and basic psychological need satisfaction in relation to students’ inter-subject variations in procrastination dimensions (Table 6). Even though, Chowdhury & Turin [60] signal that “one disadvantage of the backward elimination method is that once a variable is eliminated from the model it is not re-entered again” (p. 4) and “however, a dropped variable may become significant later in the final model”, the regression analyses were based on backward elimination method, because it “has the advantage to assess the joint predictive ability of variables as the process starts with all variables being included in the model” and “backward elimination also removes the least important variables early on and leaves only the most important variables in the model” (ibidem) [60]. Backwards elimination starts by including all selected variables into a first model and modifies models one-by-one, each time discarding the variable accounting for the least proportion of variations in the criterion, and retaining a final model with only variables significantly accounting for variations in an outcome. Also, Backward elimination was chosen, as theory indicates that variations in procrastination and other academic outcomes may depend on variations in time perspectives, psychological need satisfaction, and their interactions. “In all stepwise selection methods including all subset selection, a stopping rule or selection criteria for inclusion or exclusion of variables need to be set. Generally, a standard significance level for hypothesis testing is used”, “If the stopping rule is based on *p* values, the traditional choice for significance level is 0.05 or 0.10.” (p. 6) [60]. Lastly, we used PROCESS macro version 3.5 in SPSS 27 to test the moderating roles of basic psychological need satisfaction on the relationships between time perspectives and procrastination dimensions (Table 7).

**Table 4:** Descriptive statistics and differences in study variables according to balanced time perspective

					BTP (*n* = 469)		Not BTP (*n* = 719)			*Cohen’s*
	*M*	*SD*	Skew	Kurtosis	*M*	*SD*	*M*	*SD*	*t*	*d*
Future	3.84	0.67	-0.35	-0.15	4.00	0.55	3.73	0.72	7.23**	0.42
Past-positive	4.08	0.71	-0.79	0.50	4.19	0.55	4.01	0.78	4.48**	0.27
Present-hedonistic	3.41	0.78	-0.17	-0.15	3.09	0.61	3.61	0.81	-12.59**	0.73
Present-fatalistic	2.24	0.83	0.45	-0.18	1.97	0.63	2.41	0.90	-9.98**	0.57
Past-negative	2.93	0.93	0.14	-0.55	2.30	0.54	3.34	0.89	-25.20**	1.41
Deviation from BTP	2.13	0.78	0.50	0.09	1.60	0.50	2.47	0.73	-24.35**	1.39
Competence NS	3.77	0.70	-0.52	0.58	3.87	0.63	3.71	0.74	4.09**	0.23
General autonomy NS	3.67	0.89	-0.39	-0.25	3.79	0.80	3.59	0.94	3.81**	0.23
Academic autonomy NS	2.30	0.95	0.41	-0.50	2.24	0.93	2.33	0.96	-1.67	0.10
Relatedness NS	4.01	0.68	-0.74	0.86	4.07	0.61	3.97	0.71	2.64**	0.15
BPNS (average)	3.59	0.56	-0.30	0.48	3.65	0.49	3.55	0.60	3.31**	0.18
Decisional procrastination	2.66	0.85	0.23	-0.40	2.44	0.74	2.81	0.88	-7.83**	0.46
Implemental delay	2.98	0.86	0.08	-0.41	2.79	0.82	3.10	0.85	-6.26**	0.37
Lateness	2.24	0.77	0.45	-0.23	2.04	0.68	2.37	0.81	-7.70**	0.44
Pure procrastination	2.62	0.67	0.19	-0.35	2.42	0.60	2.76	0.69	-8.90**	0.53

*BTP* Balanced time perspective, *NS* Need satisfaction, *BPNS* Basic psychological need satisfaction; Range for all variables: 1–5, except Deviation from BTP: 0.55–4.74

**Table 5:** Pearson correlations and reliabilities of study variables

	1	2	3	4	5	6	7	8	9	10	11	12	13	14	15
1. Future	.60														
2. Past-positive	.27**	.61													
3. Present-hedonistic	-.01	.20**	.74												
4. Present-fatalistic	-.14**	.04	.17**	.60											
5. Past-negative	-.11**	-.16**	.06*	.08**	.77										
6. Deviation from BTP	-.24**	-.46**	.16**	.44**	.62**	—									
7. Competence NS	.37**	.27**	.13**	-.13**	-.19**	-.25**	.81								
8. General autonomy NS	.23**	.20**	.03	-.12**	-.16**	-.18**	.53**	.64							
9. Academic autonomy NS	.06*	.08**	.10**	.11**	.02	.01	.28**	.25**	.74						
10. Relatedness NS	.26**	.32**	.15**	-.02	-.19**	-.24**	.52**	.40**	.13**	.82					
11. BPNS (average)	.34**	.32**	.15**	-.06*	-.19**	-.25**	.85**	.72**	.52**	.76**	.86				
12. Decisional procrastination	-.32**	-.10**	.07*	.24**	.27**	.25**	-.28**	-.22**	.04	-.15**	-.23**	.70			
13. Implemental delay	-.48**	-.09**	.12**	.12**	.17**	.16**	-.24**	-.13**	-.08**	-.10**	-.20**	.49**	.76		
14. Lateness	-.48**	-.11**	.15**	.12**	.19**	.16**	-.24**	-.18**	.05	-.19**	-.21**	.47**	.53**	.67	
15. Pure procrastination	-.53**	-.12**	.15**	.19**	.25**	.23**	-.31**	-.21**	.00	-.18**	-.26**	.77**	.85**	.82**	.83

^*^p < .05; ** *p* < .01. *BTP* balanced time perspective, *BPNS* basic psychological need satisfaction, *NS* Need Satisfaction; Balanced time-perspective is not included since it is a dichotomous variable. Cronbach’s alphas reported on the diagonal

**Table 6:** Regressions of study variables on between-subject variations in procrastination dimensions

Decisional procrastination←	*B (SE)*	ß	*t*	*F*	*R*^*2*^
				46.96***	.22
Future	-.27 (.04)	-.22	-7.76***		
Present-hedonistic	.05 (.03)	.05	1.82†		
Present-fatalistic	.15 (.03)	.15	5.54***		
Past-negative	.17 (.02)	.19	7.16***		
Competence NS	-.17 (.04)	-.14	-4.22***		
Academic autonomy NS	.08 (.03)	.09	3.09**		
General autonomy NS	-.07 (.03)	-.07	-2.40*		
Implemental delay ←	*B (SE)*	ß	*t*	*F*	*R*^*2*^
				71.74***	.27
Future	-.57 (.03)	-.45	-16.55***		
Present-hedonistic	.13 (.03)	.12	4.71***		
Past-negative	.10 (.02)	.11	4.26***		
Competence NS	-.13 (.04)	-.10	-3.29**		
Academic autonomy NS	-.04 (.02)	-.05	-1.88†		
Need for relatedness	.11 (.04)	.08	2.83**		
Lateness ←	*B (SE)*	ß	*t*	*F*	*R*^*2*^
				93.20***	.28
Future	-.50 (.03)	-.43	-16.35***		
Present-hedonistic	.15 (.03)	.15	5.88***		
Past-negative	.10 (.02)	.12	4.58***		
Competence NS	-.12 (.03)	-.11	-3.73***		
Academic autonomy NS	.07 (.02)	.09	3.51***		

*NS* Need Satisfaction

^†^ = *p* < 0.1; * = *p* < .05; ** = *p* < .01; *** = *p* < .001

**Table 7:** Moderation effects of psychological need satisfaction on the relationships between time perspectives and procrastination dimensions

Decisional Procrastination ⬅	*B (SE)*	95%CI	*t*	*F*	*R*^*2*^
				62.81***	.14
Future	-.33 (.04)	-.40/-.25	-8.82***		
Competence NS	-.23 (.04)	-.30/-.16	-6.60***		
Future * Competence NS	-.09 (.04)	-.18/-.01	-2.18*		
				25.10***	.06
Present-fatalist	.25 (.03)	.19/.30	8.43***		
Academic autonomy NS	.01 (.03)	-.04/.06	.51		
Present-fatalist * Academic autonomy NS	-.06 (.03)	-.12/-.001	-1.98*		
Implemental delay ⬅	*B (SE)*	95%CI	*t*	*F*	*R*^*2*^
				123.17***	.24
Future	-.58 (.04)	-.65/-.51	-16.65***		
Competence NS	-.10 (.03)	-.16/-.03	-2.94**		
Future * Competence NS	.11 (.04)	-.19/-.03	-2.76**		
				118.17***	.23
Future	-.63 (.02)	-.69/-.56	-18.45***		
Relatedness NS	.04 (.03)	-.03/.10	1.09		
Future * Relatedness NS	-.07 (.04)	-.15/.01	-1.65†		
				118.07***	.23
Future	-.61 (.03)	-.67/-.54	-18.08***		
General autonomy NS	.03 (.03)	-.07/.02	-0.99		
Future * General autonomy NS	-.06 (.03)	-.13/.01	-1.76†		
				15.93***	.04
Past-negative	.15 (.03)	.10/.21	5.84***		
Academic autonomy NS	-.08 (.03)	-.13/-.03	-3.06**		
Past-negative * Academic autonomy NS	-.06 (.03)	-.11/-.01	-2.21*		
				14.26***	.04
Past-negative	.15 (.03)	.10/.21	5.38***		
Relatedness NS	-.08 (.04)	-.13/-.03	-2.09*		
Past-negative * Relatedness NS	-.07 (.04)	-.11/-.01	-1.78†		
Lateness ⬅	*B (SE)*	95%CI	*t*	*F*	*R*^*2*^
				122.97***	.24
Future	-.53 (.03)	-.59/-.47	-16.72***		
Competence NS	-.08 (.03)	-.14/-.03	-2.79**		
Future* Competence NS	-.07 (.04)	-.14/004	-1.85†		

*NS* Need Satisfaction

^†^ = *p* < 0.1; * = *p* < .05; ** = *p* < .01; *** = *p* < .001

Confirmatory factor analysis using AMOS 26 yielded non-equivalent chi-square distributions when comparing a model including a common latent factor against a model not including it, thus, indicating measurement discrepancies between both models. Discrepancies (D > 0.2) between standardized factor loadings resulting from comparing these two models were found only in one item from the past-positive factor (ZTPI-11: “On balance, there is much more good to recall than bad in my past”) and in two items from the past-negative factor (ZTPI-34: “It’s hard for me to forget unpleasant images of my youth”; and ZTPI-50: “I think about the bad things that have happened to me in the past”). These findings suggest that self-report questionnaires may be a sub-par way of approaching the report of judgements prompted by remembering adverse past events or of the favourable-to-adverse ratio of past events. Therefore, caution is called for in the interpretation of findings derived from these two past perspectives.

## Results

### Descriptive statistics

As Table 1 shows, the two positive time perspectives (future: 3.83 and past-positive: 4.08) yielded average scores of around 4 points; the neutral time perspective (present-hedonistic: 3.41), a score close to the centre of the scale; and the two negative time perspectives (present-fatalist: 2.24 and past-negative: 2.93) showed averaged scores, respectively, below and around the centre point of the scale. Noteworthily, both positive perspectives manifested a negative skew (mostly high scores with a long left tail, suggesting positive social desirability), whereas both negative perspectives showed a positive skew (mostly low scores with a long right tail, suggesting negative social desirability). And, interestingly, past perspectives (both positive and negative) showed higher Kurtosis, with various scores distributed more evenly, with a flatter distribution (suggesting that remembrance may be associated with less intense cognitive evaluations). Thirty-nine per cent of participants reported a balanced time perspective (BTP), and deviation from balanced time perspective (DBTP) yielded a mean of *M* = 2.13 (*SD* = 0.78), with a positive skew similar to that of the fatalistic present. Also in Table 3, it can be observed that scores for BPNS were quite high, except for the case of need for autonomy in academic learning. This suggests that satisfaction of the need for autonomy was high, but the autonomy to influence specific situations of academic learning was perceived as lower in the university setting. Furthermore, with respect to procrastination dimensions, all showed positive skewness, with the score for the lateness dimension being the highest. Only one significant gender difference was found in the study variables. Women students scored higher than men in the positive past time perspective (*M*_women_ = 4.13, *SD* = 0.71; *M*_men_ = 3.99, *SD* = 0.70; *t* = -3.449, *p* = 0.001).

### Study variables compared between students with and without a balanced time perspective (BTP)

Participants with a BTP registered more favourable levels of study variables, except for autonomy in academic learning (Table 4). Specifically, they consistently scored higher in positive time perspectives (future and past-positive), lower in negative time perspectives (present-fatalistic and past-negative), higher in overall psychological need satisfaction and lower in procrastination dimensions. As judge by Cohen’s *d*, the biggest differences between both groups involved the past-negative time perspective and the DBTP coefficient. Both differences were similar in magnitude and stood out among the other differences, suggesting that past-negative time perspective is relevant in a student’s score in DBTP. The fact that having a balanced or imbalanced time perspective accounted for differences, in both psychological need satisfaction and in procrastination dimensions, attests to the relationships between people’s individual time perspectives, contextual influences such as psychological need satisfaction, and procrastination behaviours.

### Bivariate correlations between study variables

Positive time perspectives (future and past-positive), favourably, correlated positively with BPNS and negatively with procrastination dimensions; contrarily, negative time perspectives (present-fatalistic and past-negative) showed adverse relationships, as they were associated negatively with BPNS and positively with procrastination dimensions (see Table 5). Deviation from balanced time perspective also had a relationship pattern with BPNS and procrastination similar to that of negative time perspectives (thus, adverse): negative relationships with BPNS and positive ones with procrastination. And, lastly, the present-hedonistic time perspective correlated positively and moderately both with BPNS and procrastination dimensions.

Also in Table 5, it can be observed that BPNS was negatively associated with procrastination dimensions, except for need for autonomy in academic learning (Academic autonomy), which, as mentioned above, was consistently scored lower, given that participants had less control over the way in which academic learning activities were conducted. The reliability coefficients of all the scales were satisfactory (see diagonal of Table 5) as determined by the reference threshold for Chronbach’s alpha for factors with few indicators (*α* > 0.60; e.g., [51]).

### Regression analyses for variations in procrastination dimensions accounted for by study variables

The regression analyses (Backward Method) of time perspectives and BPNS on students’ inter-subject variations in procrastination dimensions (see Table 6) showed that between-subject variations in these dimensions were explained by study variables. Firstly, 22% of variations in decisional procrastination was explained by time perspectives (except past-positive) and psychological need satisfaction (except relatedness); the strongest (favourable) negative correlate of decisional procrastination were future time perspective and competence need satisfaction, and the strongest (adverse) positive correlate of decisional procrastination variation was past negative. Furthermore, 27% of variations in implemental delay was explained by time perspectives (future, present-hedonist and past negative) and BPNS (competence and autonomy need satisfaction), the strongest correlate was future (favourable negative association with implemental delay). Lastly, 28% of variations in lateness was explained by time perspectives (future, present-hedonist, and past-negative) and BPNS (competence and academic autonomy need satisfaction), the strongest correlate being future (favourable negative relationship with lateness) (see Table 6). Noteworthily, the valence of every relationship between a procrastination dimension and another study variable (time perspective or BPNS) was as expected by theory, except for the fact that academic autonomy need satisfaction (being able to influence or decide on aspects of the learning activities specifically) positively correlated with variations in decisional procrastination and lateness (albeit in small proportions), suggesting that allowing students to make decisions that influence learning designs could in some cases be linked with adverse academic outcomes.

### Moderating role of psychological need satisfaction on the relationships between time perspectives and procrastination dimensions

With regards to the moderating role of psychological need satisfaction on the relationships between time perspectives and procrastination dimensions (see Table 7), eight moderations were observed – in line with theory – albeit mostly with modest significance levels, except for that of perceived competence strengthening the negative relationship between future time perspective and implemental delay.

**Fig. 1:**
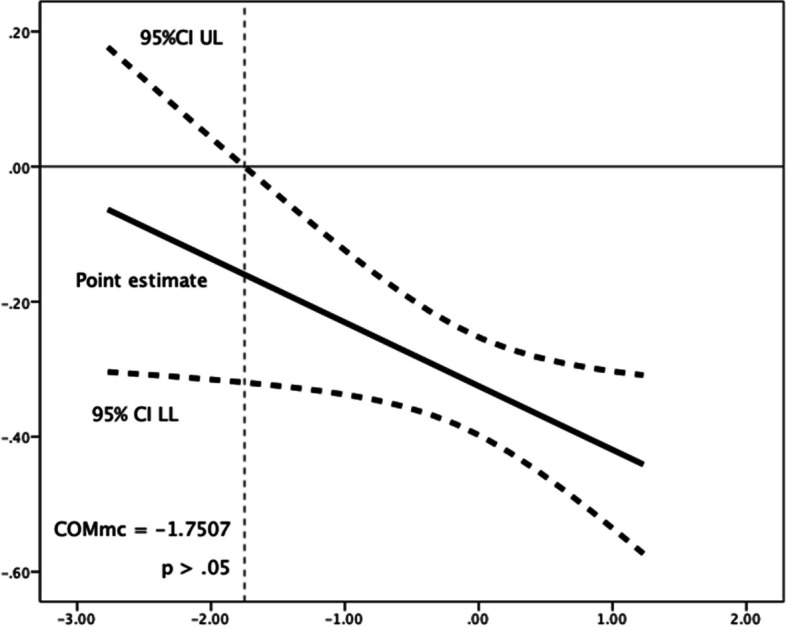
Johnson-Neyman Plot for conditional association of future time perspective and decisional procrastination, moderated by (mean-centered) competence need satisfaction**.** The association ceases to be significant (*p* < .05) below a mean-centered competence need satisfaction score of -1.7507

**Fig. 2:**
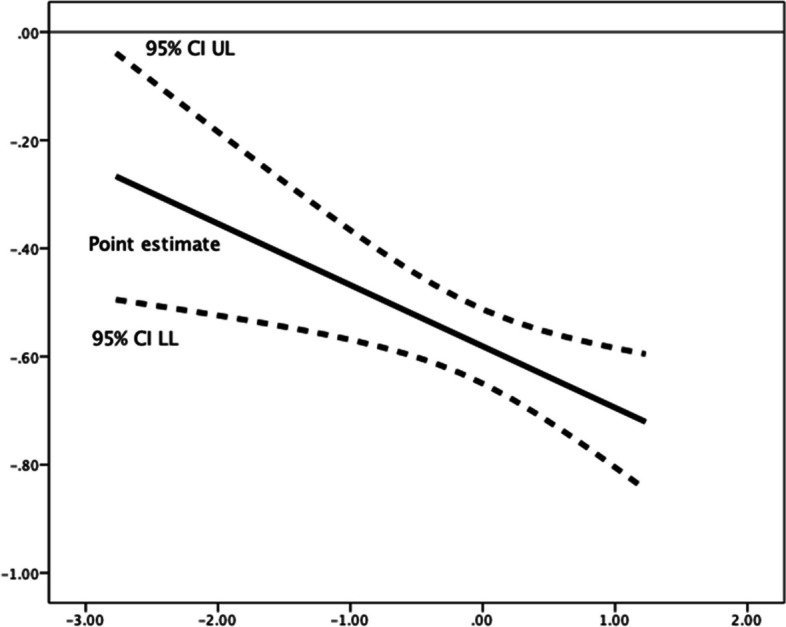
Johnson-Neyman Plot for conditional association of future time perspective and implemental delay, moderated by (mean-centered) competence need satisfaction**.** There are no statistical significance transition points within the observed range of the moderator found using the Johnson-Neyman method

**Fig. 3:**
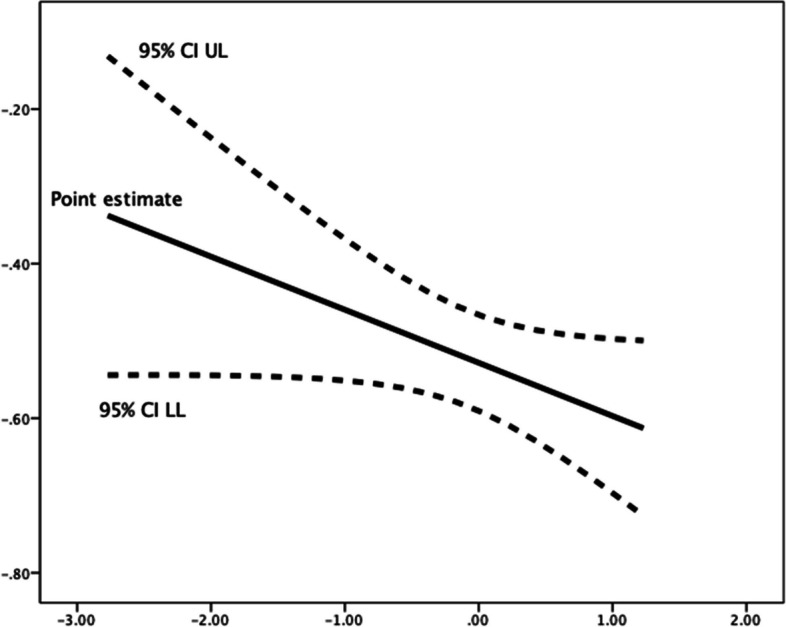
Johnson-Neyman Plot for conditional association of future time perspective and lateness, moderated by (mean-centered) competence need satisfaction**.** There are no statistical significance transition points within the observed range of the moderator found using the Johnson-Neyman method

Competence need satisfaction moderated the relationships between future time perspective and each of the three procrastination dimensions. Greater scores in perceived competence were linked with an increase in the (favourable) negative relationships of future time perspective, respectively, with decisional procrastination (*R*^*2*^ change = 0.004, *F*(1,1184) = 4.739, *p* = 0.030), implemental delay (*R*^*2*^ change = 0.005, *F*(1,1184) = 7.621, *p* = 0.006), and lateness (*R*^*2*^ change = 0.002, *F*(1,1184) = 3.416, *p* = 0.065). Use of the Johnson-Neyman technique for determining regions of significance showed that below a mean-centred competence need satisfaction score of -1.7507 (1.85% of participants), the relationship of future time perspective with decisional procrastination failed to reach (*p* < 0.05) significance levels (see Table 7 and Fig. 1); in the cases of implemental delay (Fig. 2) and lateness (Fig. 3), their negative associations with future time perspective were significant (*p* < 0.05) along all the spectrum of competence need satisfaction.

**Fig. 4:**
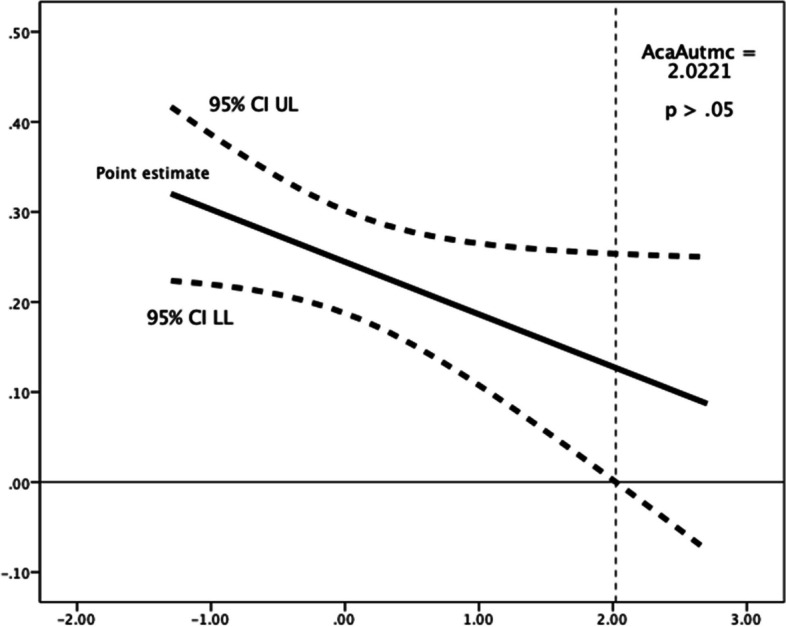
Johnson-Neyman Plot for conditional association of present-fatalist time perspective and decisional procrastination, moderated by (mean-centered) academic autonomy need satisfaction. The association ceases to be significant (*p* < .05) above a mean-centered academic autonomy need satisfaction score of 2.0221

**Fig. 5:**
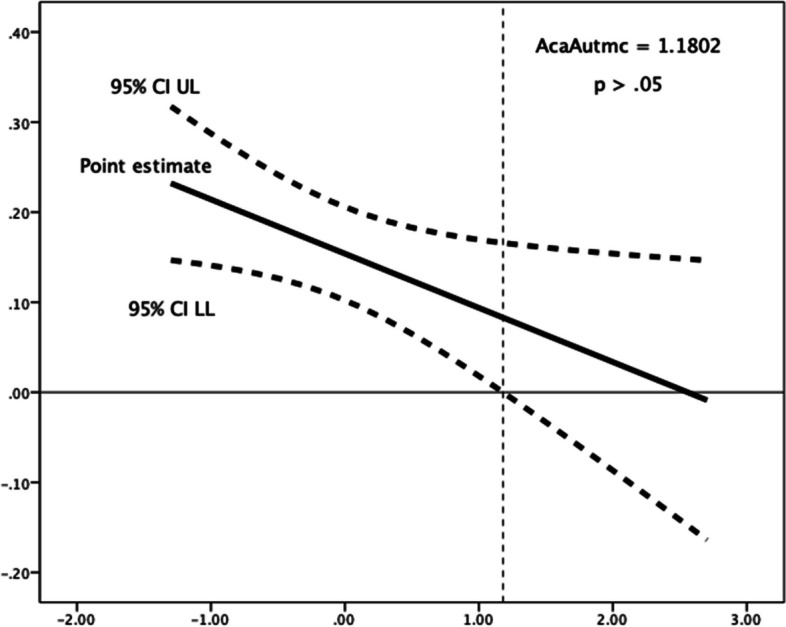
Johnson-Neyman Plot for conditional association of past-negative time perspective and implemental delay, moderated by (mean-centered) academic autonomy need satisfaction. The association ceases to be significant (*p* < .05) above a mean-centered academic autonomy need satisfaction score of 1.1802

Academic autonomy need satisfaction (as in students perceiving to have the possibility of participating in the design of academic course characteristics) moderated the relationships between present-fatalistic time perspective and decisional procrastination (*R*^*2*^ change = 0.003, *F*(1,1184) = 3.926, *p* = 0.048), and between past-negative time perspective and implemental delay (Fig. 5: *R*^*2*^ change = 0.004, *F*(1,1184) = 4.861, *p* = 0.028). In line with the theory, the two aforementioned time perspectives are considered negative. Higher the levels of perceived satisfaction of the need for academic autonomy were associated with weakened (adverse) positive relationships of present-fatalist and past-negative time perspectives, respectively, with decisional procrastination and implemental delay. In this case, use of the Johnson-Neyman technique showed that the (adverse) positive relationship of present-fatalistic time perspective with decisional procrastination failed to reach (*p* < 0.05) significance above a mean-centred score of 2.0221 in satisfaction of the need for academic autonomy (Fig. 4). Also, use of the Johnson-Neyman technique showed that when a mean-centred score in satisfaction of the need for academic autonomy exceeded 1.1802, the (adverse) positive relationship of past-negative time perspective with implemental delay failed to reach (*p* < 0.05) significance levels (Fig. 5), suggesting a favourable role of academic autonomy need satisfaction (linked negatively with implemental delay), notwithstanding the fact that academic autonomy was found to be positively associated with decisional procrastination and lateness at a bivariate level.

**Fig. 6:**
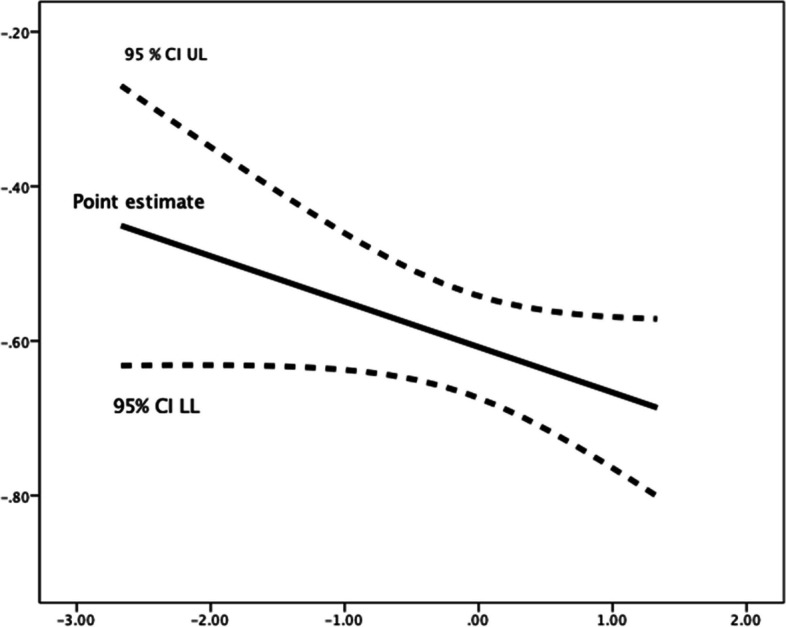
Johnson-Neyman Plot for conditional association of future time perspective and implemental delay, moderated by (mean-centered) general autonomy need satisfaction. There are no statistical significance transition points within the observed range of the moderator found using the Johnson-Neyman method

General autonomy need satisfaction (two items regarding being able to express opinions or make decisions, in general) moderated the relationship between future and implemental delay (*R*^*2*^ change = 0.002, *F*(1,1184) = 3.083, *p* = 0.079, see Fig. 6). Higher levels of perceived satisfaction of the need for general autonomy (2-items regarding general autonomy to express opinions), were associated with increased (favourable) negative relationships of future time perspective with implemental delay (see Table 5).

**Fig. 7:**
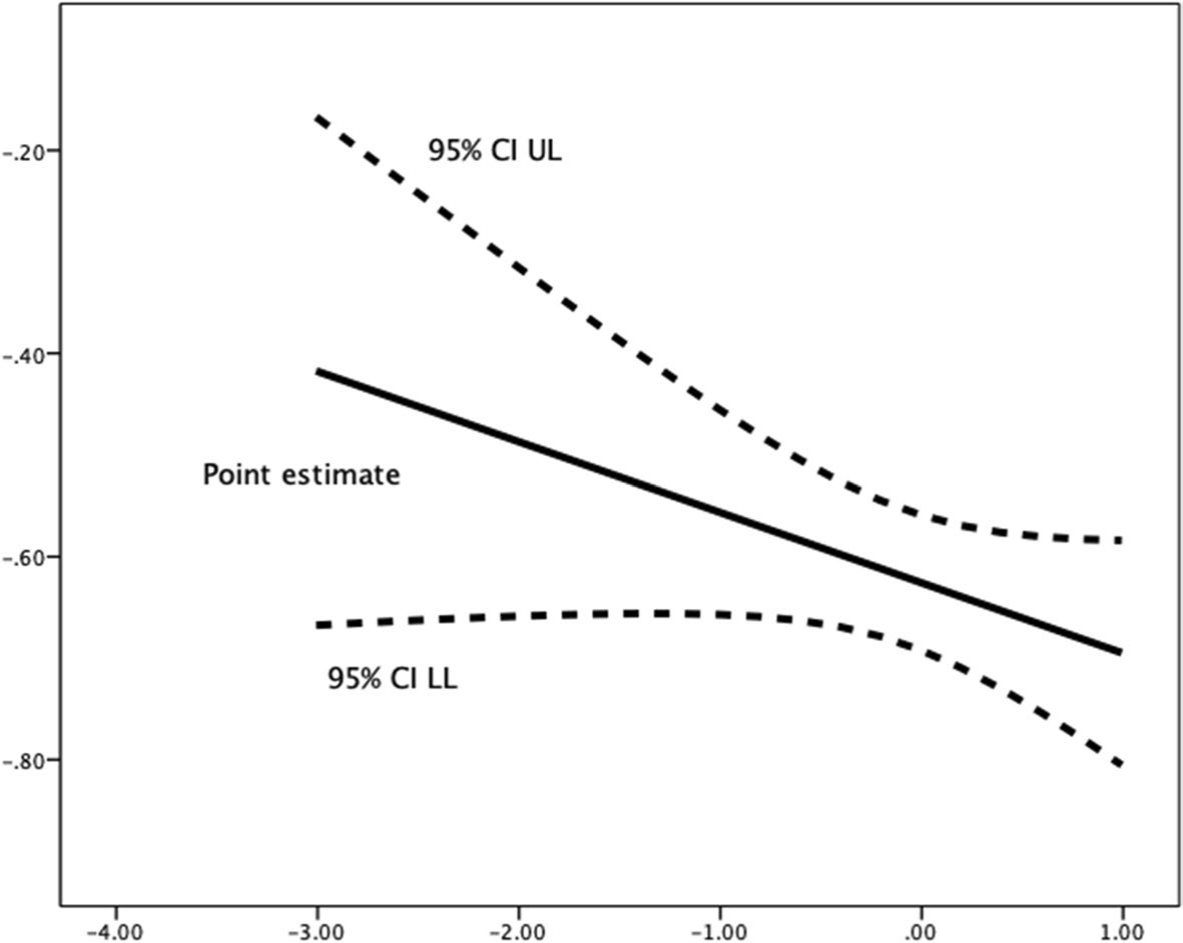
Johnson-Neyman Plot for conditional association of future time perspective and implemental delay, moderated by (mean-centered) relatedness need satisfaction. There are no statistical significance transition points within the observed range of the moderator found using the Johnson-Neyman method

**Fig. 8:**
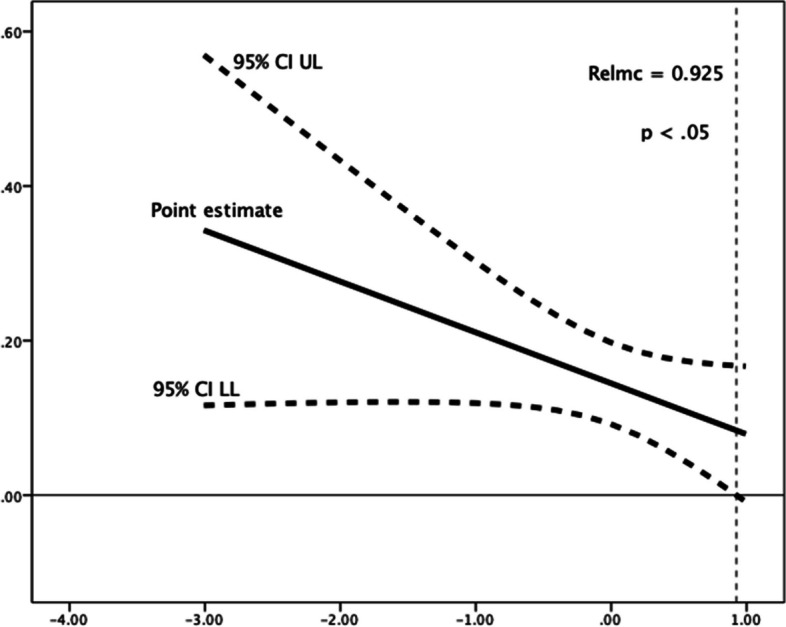
Conditional association of past-negative time perspective and implemental delay, moderated by (mean-centered) relatedness need satisfaction. The association ceases to be significant (*p* < .05) above a mean-centered relatedness need satisfaction score of 0.925

Lastly, relatedness need satisfaction moderated the relationships between implemental delay and two of the time perspectives: future (*R*^*2*^ change = 0.002, *F*(1,1184) = 2.791, *p* = 0.099) and past-negative (*R*^*2*^ change = 0.003, *F*(1,1184) = 3.170, *p* = 0.075). Higher levels of relatedness need satisfaction, were associated with an increase in the (favourable) negative relationship of future time perspective with implemental delay (Fig. 7); and with a decrease in the (adverse) positive relationship of past-negative time perspective with implemental delay, which rendered not significant (at a *p* < 0.05 significance level, see Fig. 8) above a mean-centred relatedness need satisfaction score of 0.925.

## Discussion

Procrastination is a problem whose impact on students can extend to other areas of everyday activity and also later stages of life. Research is needed to support reflection on classroom intervention strategies that may serve in tackling procrastination today and in preventing its persistence in the future. In fact, the research on procrastination in relation to time perspective factors and basic psychological needs places this problem at the meeting point of individual and contextual variables – whose explanations may conjointly account for the complexity of this problem. The present study focused both on the individual, given that time perspectives can be defined as a person’s attitude to an object (time) at three moments (present, past, future); and on contextual aspects, because the satisfaction of basic psychological needs for competence, autonomy, and relatedness is facilitated or made difficult by social contexts. Both sides of this phenomenon – individual and contextual – make patent the need for an integrated perspective regarding self-regulatory problems expressed in procrastination, and which, in a deep sense, are intertwined with the satisfaction of basic psychological human needs.

### Associations between time perspectives, psychological need satisfaction, and procrastination dimensions

It is in this confluence of individual and contextual variables that the present work contributes to previous research on procrastination. The framework of time perspectives and its connection to the competence of time management finds a revealing problem in the phenomenon of procrastination. The combination of attitudes towards present, past and future – in their modalities of balanced time perspective and deviation from balanced time perspective – shows that positive attitudes towards the general experience of time (i.e. a balanced time perspective) are related to fewer delays in what needs to be done (the opposite of those who have a deviation from balanced time perspective).

H_1_ supported by the results of previous research [3, 12] was fully substantiated, given that future, past-positive and balanced time perspectives were negatively associated with all procrastination measures. In the case of the present-hedonistic perspective, it positively correlated with decisional procrastination, implemental delay, lateness and pure procrastination, corroborating previous findings [18]. Likewise, H_2_ was also substantiated, given that the perspectives of the fatalistic present, negative past and deviation from balanced time were positively associated with procrastination dimensions.

Regarding a context that promotes basic psychological need satisfaction, it is argued that this satisfaction may be associated with lesser manifestations of procrastination. Specifically, regarding substantiation for H_3_, the satisfaction of the needs for competence, autonomy and relatedness was, as expected, negatively associated with procrastination dimensions. Academic autonomy need satisfaction in particular showed only one significant – as expected, negative – correlation with a procrastination dimension, which was implemental delay (Table 5). These results make clear that the more BPNS observed, the fewer the manifestations of procrastination.

### Differences in study variables according to balanced time perspective

In addition to what was posited in the hypotheses, it is worth noting the differences observed according to whether or not a BTP was present. These differences were significant in practically all the variables considered (with the exception of need for autonomy), i.e. participants who reported a balanced time perspective consistently scored higher in positive time perspectives, lower in negative time perspectives, higher in overall BPNS, and lower in procrastination. Thus, procrastinators do not have a balanced time perspective while, in contrast, individuals with a prevalence of future, past-positive and present-hedonistic orientation together with positive competence, positive autonomy and relatedness need satisfaction have a balanced time perspective. Taken as a whole, our results support previous evidence regarding the balance or imbalance of time perspectives [16], introducing a novel element: the relationships between balanced time perspective, BPNS and procrastination.

### Variations in procrastination dimensions accounted for by time perspectives and psychological need satisfaction

As regards the most salient associations of procrastination variations with other study variables, future time perspective stood out as the most relevant (favourable) negative correlate of variations in all three procrastination dimensions. In turn, past negative time perspective had a positive (adverse) relationship with variations in decisional procrastination, and the opposite was true for competence need satisfaction which had a (favourable) negative relationship with decisional procrastination. These results resonate with the robust negative correlations observed between procrastination dimensions and future time perspective and competence need satisfaction; and with the fact that these two variables were positively associated with each other and negatively with past negative time perspective. Put together these findings signal that a combination of individual and contextual aspects which seems opposed to procrastination tendencies would result from high future and low past negative time perspectives and high competence need satisfaction.

Interestingly, academic autonomy need satisfaction (three items about influencing or deciding on learning activities) has positive (adverse) relationships with decisional procrastination and lateness, accounting for small proportions in both their variations, signalling that teachers may need to look out for the potential links between allowing students to make decisions regarding their learning activities and adverse learning outcomes.

### Moderating roles of psychological need satisfaction on the relationship between time perspectives and procrastination dimensions

With regards to H_4A_ and H_4B_, these were substantiated given that it was possible to observe eight moderations of psychological need satisfaction on relationships between time perspectives and procrastination dimensions, all in the expected directions. The (adverse) positive relationships of negative time perspectives (present fatalistic and past negative) with procrastination dimensions, depicted by steep ascending line diagrams, were weakened given high levels of satisfaction of specific psychological needs; and the (favourable) negative relationships of positive time perspectives (future and past positive), depicted by descending line diagrams, were strengthened (steepened) given high levels of psychological need satisfaction. In particular, competence need satisfaction moderated the relationships between future perspective and all three procrastination dimensions, in the sense that the higher the competence need satisfaction the stronger (favourable) negative relationships with implemental delay, decisional procrastination and lateness. Other aspects worth highlighting were, on the one hand, that the satisfaction of the need for autonomy moderated the positive relationships of present-fatalistic and past-negative time perspectives with decisional procrastination and implemental delay, respectively; and, on the other hand, the satisfaction of the need for autonomy, also moderated the positive relationship of the fatalistic present with decisional procrastination and of negative past with implemental delay. These moderations suggest that contextual conditions such as BPNS may strengthen or weaken the relationships that other variables such as time perspectives may have with procrastination dimensions (in line with [23, 26]).

### Contributions to instrumentation

Taking into account the value of this research in the empirical analysis of procrastination, improvements were made in the instruments used for measuring time perspectives, basic psychological need satisfaction and procrastination. The performance of CFAs contributed to the robustness of the three instruments for Spanish-speaking participants, which may lead to further research with higher levels of validity in these populations. In the case of the time perspectives, the proposal of a 17-item inventory is part of recent initiatives aimed at improving the structure and functioning of the ZTPI, while maintaining the original factors [61]. Regarding basic psychological need satisfaction, the behaviours linked to the two aspects of autonomy (the general need for autonomy satisfaction and the specific need for autonomy satisfaction in academic contexts) deserve deeper examination in future studies – especially given the importance of the need for competence during the university stage (i.e. prior to incorporation into the labour market). Regarding the measurement of pure procrastination, the analyses carried out offer a better definition of the instrument used to analyse this problem in our context.

### Limitations of the study

The lines of action described above do not obviate the limitations of this research, due to cross-sectional design limits the possibility of drawing robust causal inferences, and due to the convenience sampling method limits the possibility of generalization to the population and of gender or degree-based comparisons, which will have to be tackled by replication. The relationships observed and the contributions made by one of the instruments require further studies with other samples and procedures different from those used here. Specifically, the successive adaptations of the ZTPI signal that this measurement instrument requires multiple analyses of its structure and functioning. With regard to the measure of procrastination, the relationships between its factors must go hand in hand with the theoretical conceptions from which this construct is investigated, in addition to being mathematically sound. The present study was not preregistered.

## Conclusions

The reported findings (specifically, the moderating role of BPNS in the relationships between time perspectives and procrastination) contribute, as value added to the research about procrastination, that basic psychological need satisfaction can play a relevant role in catalysing the favourable (negative) relationships of positive time perspectives with procrastination and also attenuate the adverse (positive) relationships of negative time perspectives with procrastination. In this regard, contextual interventions fostering enhanced levels of perceived autonomy, competence, and relatedness are strong candidates for use by policy makers, pedagogues, teachers, coaches and other professionals who are interested in helping their students, staff or practitioners cultivate conditions that may counteract procrastination tendencies.

In order to tackle procrastination among university students, our results have brought to light two principal lines of action. On the one hand, a future perspective should be promoted along the lines of the original proposal [14]. Up to now, the future – in relation to procrastination – has been treated as part of a general framework [62] measured with alternative instruments to the ZTPI [63]. This makes it difficult to relate the dynamics of this aspect of time to the past and the present. It is worth noting that our results show the importance of not only counteracting negative views of the past but also the incidence in everyday life of present-fatalistic and present-hedonistic perspectives. And on the other hand, it is necessary to focus on the need for autonomy in its twofold aspect: as a psychological need and as a transversal competence in the educational world of work. In this sense, it is worth keeping in mind that, in interventions to tackle procrastination, the findings related to BPNS are of particular importance, an approach whose emphasis on self-regulation plays a fundamental role in interventions designed to alleviate this problem [64].

There is also a need to continue investigating the variables that determine the limits of the individual and the contextual in procrastination – the variables that explain it and the consequences derived from this problem. Regarding individual factors, attitudes towards time – and their corresponding balances or imbalances – must dialogue with other individual traits whose instrumental expression is associated with procrastination. In this way, the experience of temporality is assimilable to a characteristic of the human that is challenged by the factors it encounters within contexts. Regarding these contextual factors, the satisfaction of the basic psychological needs studied here entails the use of classroom strategies that promote such satisfaction.

In sum, contributing to the design and implementation of intervention programmes that understand, instrumentalise and intervene in the relationships linking the problem of procrastination, general attitudes towards time and the satisfaction of basic psychological needs would offer a promising research scenario to be explored without delay.

## Data Availability

No datasets were generated or analysed during the current study.

## Abbreviations

BPNS: Basic psychological need satisfaction
BTP: Balanced time perspective
CFA: Confirmatory factor analyses
DBTP: Deviation from balanced time perspective
SDT: Self-determination Theory
ZTPI: Zimbardo Time Perspective Inventory
